# Mitochondrial uncoupling has no effect on microvascular complications in type 2 diabetes

**DOI:** 10.1038/s41598-018-37376-y

**Published:** 2019-01-29

**Authors:** Lucy M. Hinder, Kelli M. Sas, Phillipe D. O’Brien, Carey Backus, Pradeep Kayampilly, John M. Hayes, Cheng-mao Lin, Hongyu Zhang, Sumathi Shanmugam, Amy E. Rumora, Steven F. Abcouwer, Frank C. Brosius, Subramaniam Pennathur, Eva L. Feldman

**Affiliations:** 1 Department of Neurology, University of Michigan, Ann Arbor, MI 48109 USA; 2 Division of Nephrology, Department of Internal Medicine, University of Michigan, Ann Arbor, MI 48109 USA; 3 Department of Ophthalmology and Visual Sciences, University of Michigan, Ann Arbor, MI 48105 USA; 4 Departments of Molecular and Integrative Physiology, University of Michigan, Ann Arbor, MI 48109 USA

## Abstract

Diabetic peripheral neuropathy (DPN), diabetic kidney disease (DKD), and diabetic retinopathy (DR) contribute to significant morbidity and mortality in diabetes patients. The incidence of these complications is increasing with the diabetes epidemic, and current therapies minimally impact their pathogenesis in type 2 diabetes (T2D). Improved mechanistic understanding of each of the diabetic complications is needed in order to develop disease-modifying treatments for patients. We recently identified fundamental differences in mitochondrial responses of peripheral nerve, kidney, and retinal tissues to T2D in BKS-*db/db* mice. However, whether these mitochondrial adaptations are the cause or consequence of tissue dysfunction remains unclear. In the current study BKS-*db/db* mice were treated with the mitochondrial uncoupler, niclosamide ethanolamine (NEN), to determine the effects of mitochondrial uncoupling therapy on T2D, and the pathogenesis of DPN, DKD and DR. Here we report that NEN treatment from 6–24 wk of age had little effect on the development of T2D and diabetic complications. Our data suggest that globally targeting mitochondria with an uncoupling agent is unlikely to provide therapeutic benefit for DPN, DKD, or DR in T2D. These data also highlight the need for further insights into the role of tissue-specific metabolic reprogramming in the pathogenesis of diabetic complications.

## Introduction

In the United States, 30 million adults^1^ and 200,000 youth^2^ have type 2 diabetes (T2D), and this number is expected to double by 2050^1,3^. The complications of diabetes are common and disabling. A high incidence of cardiovascular disease and stroke, commonly classified as diabetic macrovascular disease, contributes to significant patient morbidity and mortality^1^. More common than these macrovascular complications are the microvascular complications of diabetes, which are disabling and poorly understood. Diabetic peripheral neuropathy (DPN), diabetic kidney disease (DKD), and diabetic retinopathy (DR) contribute to significant morbidity and mortality, and the incidence of these diabetic microvascular complications is increasing with the diabetes epidemic^4–6^.

DPN is the most common diabetic complication, affecting approximately half of all diabetics. DPN is characterized by progressive loss of peripheral nerve function (distal extremities affected first), with pain and eventual loss of sensation^4^. DPN is the leading cause of diabetes-related hospital admissions in the US, with an estimated economic burden of $20 billion^7,8^. DKD, characterized by albuminuria and compromised glomerular filtration, affects ~30% of all diabetics and is the leading cause of end-stage renal disease^5^. Similarly, DR occurs in approximately 35% of diabetics^6^ and is one of the leading causes of moderate and severe vision loss worldwide^9^.

Despite the prevalence of these diabetic complications, current therapies minimally impact the development and progression of T2D end-organ damage in nerve, kidney, and retina. Moreover, therapies targeting a specific pathway in one complication may exacerbate another^10,11^. Improved mechanistic understanding of each of the diabetic complications is needed, with the goal of developing disease-modifying treatments for patients.

Using a combination of transcriptomics, lipidomics, and *in vivo* fluxomics, we recently identified tissue-specific lipid signatures^12^ and changes in mitochondrial metabolism^13^ in peripheral nerve, kidney and retina in the BKS-*db/db* mouse model of T2D. In parallel, we reported that perturbations of the metabolic syndrome in *db/db* mice lead to distinct differences in each end-organ^13^, associated with differential transcriptional regulation of mitochondrial lipid and oxidative pathways in the three different tissues^10^. These data highlight fundamental differences in mitochondrial responses within the nerve, kidney and retina, but whether these mitochondrial adaptations are the cause or consequence of tissue dysfunction remains unclear.

**Figure 1:**
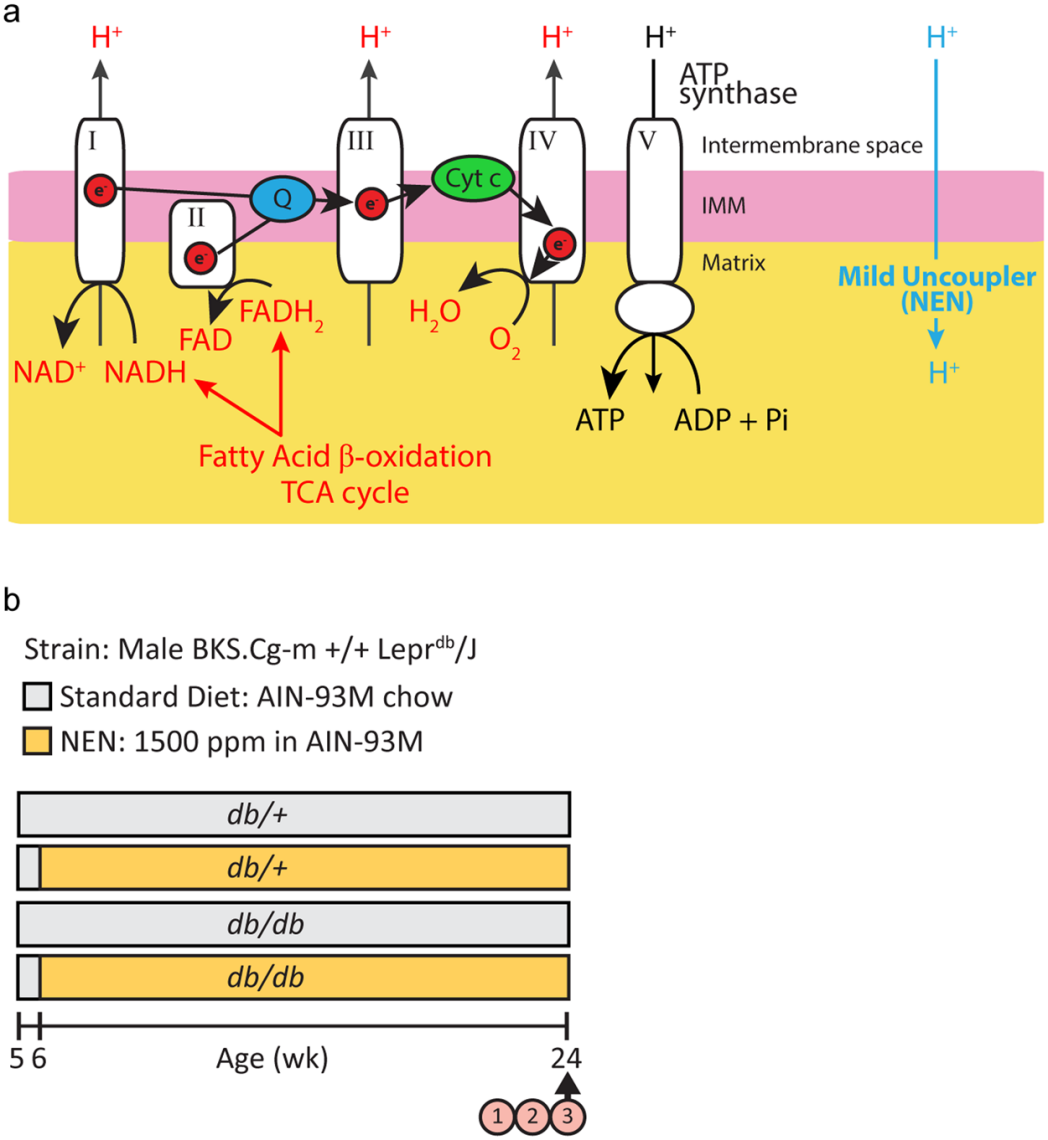
NEN-mediated mitochondrial uncoupling & Study design. (**a**) NADH and FADH_2_ reducing equivalents from upstream mitochondrial metabolism enter the mitochondrial respiratory chain. As they travel along the respiratory chain, complexes I – IV pump protons from the mitochondrial matrix to the intermembrane space. ATP synthase/complex V uses this proton gradient to generate ATP. NEN allows protons to pass back into the mitochondrial matrix, bypassing ATP synthase-mediated ATP production. Thus, mitochondrial respiration is uncoupled from energy production. This “release” of the proton gradient allows more reducing equivalents from NADH and FADH_2_ to enter the respiratory chain, increasing upstream fatty acid oxidation and TCA cycling (represented by red font). (**b**) Study design. 1. Metabolic phenotyping, 2. diabetic peripheral neuropathy, diabetic kidney disease, and diabetic retinopathy phenotyping, 3. dorsal root ganglion neuron mitochondrial coupling efficiency. NEN, niclosamide ethanolamine.

In healthy mitochondria, reducing equivalents from mitochondrial fatty acid oxidation and the tricarboxylic acid (TCA) cycle enter the mitochondrial respiratory chain as electrons donated from NADH and FADH_2_ (Fig. 1a). These electrons are shuttled along the electron transport chain and are eventually donated to molecular oxygen, forming H_2_O in a process known as mitochondrial respiration. Complexes I - IV in the inner mitochondrial membrane use free energy from the electrons to pump protons from the mitochondrial matrix to the intermembrane space. This creates a proton gradient across the mitochondrial membrane, which ATP synthase/complex V uses to generate ATP. Uncoupling proteins and drugs allow protons to pass back into the mitochondrial matrix, bypassing ATP synthase-mediated ATP production^14,15^. Thus, mitochondrial respiration is uncoupled from energy production. This “release” of the proton gradient allows more reducing equivalents from NADH and FADH_2_ to enter the respiratory chain, potentially increasing upstream fatty acid oxidation and TCA cycling^14,15^ (Fig. 1a). Using the liver-targeted, mild uncoupling drug, niclosamide ethanolamine (NEN), Tao *et al*.^16^ reported that such uncoupling increased metabolism in the liver of high fat-fed mice^16^, and that this increase was associated with decreased liver lipid accumulation, decreased body weight, and improved glycemic control. This same group also reported that uncoupling with NEN improved glycemic control in *db/db* T2D mice^16^. Thus, mild mitochondrial uncoupling can increase substrate catabolism and improve the metabolic signature of T2D phenotypes.

A role for mitochondrial uncoupling in the pathogenesis of diabetic microvascular complications has also been suggested. Differential regulation of endogenous uncoupling proteins is implicated in mitochondrial dysfunction associated with DPN^17^, DKD^13,18–23^ and DR^24–26^, however, mechanistic studies have largely involved type 1 diabetes (T1D) and those investigating all three tissues in a single model or setting are lacking. The current study was designed to address this scientific gap by investigating the effects of NEN uncoupling therapy on DPN, DKD, and DR in BKS-*db/db* mice.

We report that NEN treatment of *db/db* mice from 6–24 wk of age had little effect on the development of T2D and diabetic complications. Our data suggest that directly targeting mitochondria with NEN is unlikely to provide therapeutic benefit for DPN, DKD or DR in T2D. Our findings also highlight the need for further insights into the role of tissue-specific metabolic reprogramming in the pathogenesis of diabetic microvascular complications.

## Results

### Metabolic phenotyping

**Figure 2:**
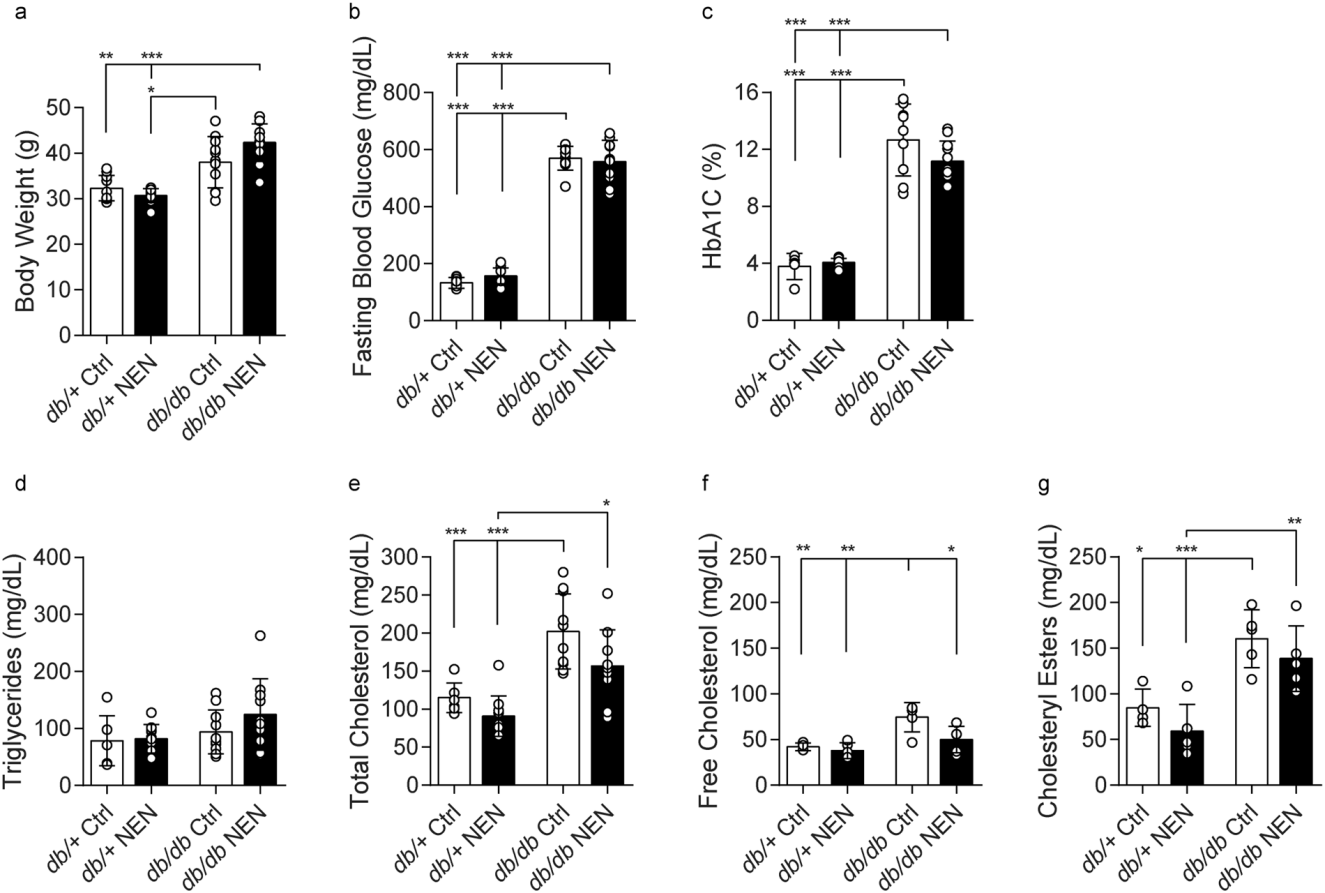
Metabolic phenotyping. (**a**) Body weight, (**b**) fasting blood glucose, (**c**) glycated hemoglobin, (**d**) total fasting plasma triglycerides, (**e**) total fasting plasma cholesterol, (**f**) free cholesterol and (**g**) cholesteryl esters were measured at 24 wk. Ctrl, mice fed a standard diet; NEN, mice fed niclosamide ethanolamine chow. *p < 0.05, **p < 0.01, ***p < 0.001.

The BKS-*db/db* mouse represents a robust model of T2D^27–33^. These mice lack a functional leptin receptor, conferring impaired satiety signaling, resulting in T2D with hyperphagia, obesity, hyperglycemia, and dyslipidemia. We first examined the effect of NEN treatment on metabolic parameters of T2D in the BKS-*db/db* animals (Fig. 1b). At 24 wk, *db/db* mice were heavier and developed hyperglycemia (Fig. 2b,c; p < 0.0001 for both) and hypercholesterolemia (Fig. 2e; p = 0.0004) compared with age-matched *db/*+ controls. Circulating triglycerides were unchanged in *db/db* mice compared to age-matched *db*/+ controls (Fig. 2d). Notably, *db/db* body weight plateaued from 12–18 wk of age (Supplementary Fig. S1), with weight loss from 20–24 wk. Although NEN-treated *db/db* mice were significantly heavier than *db/db* mice during this plateau phase, NEN treatment did not prevent the age-related *db/db* weight loss (Supplementary Fig. S1).

There was no significant effect of NEN treatment on glycemia or triglycerides (Fig. 2a,b-d). There was a trending NEN treatment effect on *db/db* total cholesterol (23% decrease, p = 0.065, *db/db* NEN vs. *db/db* Ctrl), which led us to quantify changes in free- and esterified-cholesterol (comprising total cholesterol) (Fig. 2f,g). We discovered an increase in both free cholesterol (p = 0.0063) and cholesterol esters (p = 0.0101) in *db/db* mice, with NEN treatment lowering free cholesterol in *db/db* mice (33% decrease, p = 0.0299, *db/db* NEN vs. *db/db* Ctrl). Notably, NEN treatment had no significant effect on any metabolic parameters in *db/*+ nondiabetic control mice.

In summary, BKS-*db/db* mice developed classic features of T2D, with a small, yet significant, treatment effect on free cholesterol.

### Diabetic peripheral neuropathy phenotyping

The BKS-*db/db* mouse develops symptoms of DPN^28,34^ from as early as 6 wk of age^32^. We next examined the effects of NEN treatment on large fiber function (electrophysiology testing of motor and sensory NCVs), small fiber function (nocifensive behavior testing of hind paw withdrawal latencies from a painful thermal stimulus), and small fiber pathology [intraepidermal nerve fiber density (IENFD) in hind paw footpads] at 24 wk of age.

**Figure 3:**
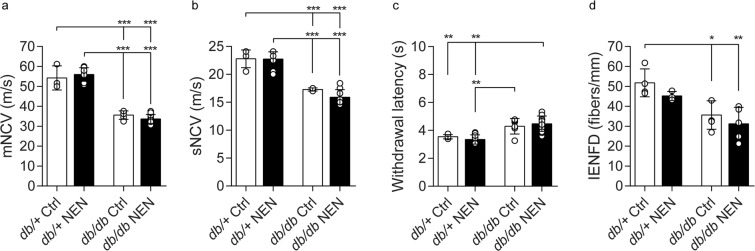
Diabetic Peripheral Neuropathy phenotyping. (**a**) Sciatic motor NCV, (**b**) sural sensory NCV, (**c**) hind paw withdrawal latency, and (**d**) intraepidermal nerve fiber density were measured at 24 wk. *p < 0.05, **p < 0.01, ***p < 0.001. NCV, nerve conduction velocity.

At 24 wk, *db/db* mice had slower motor NCV (Fig. 3a; p < 0.0001) and slower sensory NCV (Fig. 3b; p < 0.0001) compared with age-matched *db*/+ controls, indicating large fiber dysfunction. NEN treatment did not significantly modify the *db/db* decrease in NCVs (Fig. 3a,b). At 24 wk, there was a trend in increased hind paw latency among the *db/db* mice but it did not reach statistical significance (Fig. 3c), however IENFD was decreased (Fig. 3d; p = 0.0194), indicating loss of small fibers and degeneration of distal sensory nerve fibers. NEN treatment did not significantly modify either withdrawal latency or IENFD.

In summary, BKS-*db/db* mice developed deficits in large fiber function, and degeneration of distal small nerve fibers compared with *db*/+ control mice. NEN treatment did not prevent these *db/db* phenotypes.

### Diabetic kidney disease & diabetic retinopathy phenotyping

By 16 wk of age the BKS-*db/db* mouse develops compromised renal function (polyuria, elevated urinary albumin/creatinine ratio [ACR]), significant renal structural pathology (glomerular hypertrophy and mesangial sclerosis)^35,36^, and compromised visual function with retinal neurodegeneration^30,31^. We next examined whether NEN treatment could modify DKD and DR at 24 wk.

**Figure 4:**
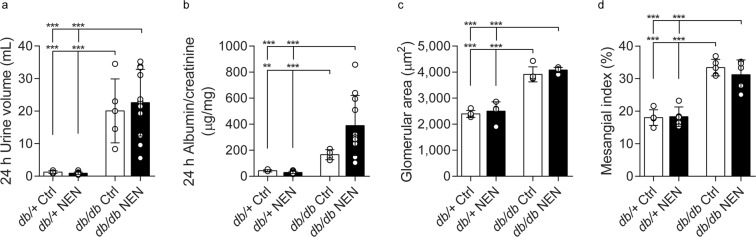
Diabetic Kidney Disease phenotyping. (**a**) 24 h urine volume, (**b**) 24 h albumin/creatinine ratio, (**c**) glomerular area, and (**d**) mesangial index were measured at 24 wk. **p < 0.01, ***p < 0.001.

As expected, *db/db* mice exhibited the hallmarks of DKD, with polyuria, increased ACR, glomerular hypertrophy, and mesangial sclerosis (Fig. 4, p < 0.0001, *db/db* Ctrl vs. *db*/+ Ctrl for all measures, except p = 0.0011 for ACR). NEN-treated *db/db* mice did not show any improvement in DKD measures. In fact, there was a trend for a worsening of the ACR in NEN-treated *db/db* mice (p = 0.053, *db/db* NEN vs. *db/db* Ctrl). See Supplementary Fig. S2 for urinary albumin, urinary creatinine, and PAS-positive glomerular area data, from which the above were calculated.

**Figure 5:**
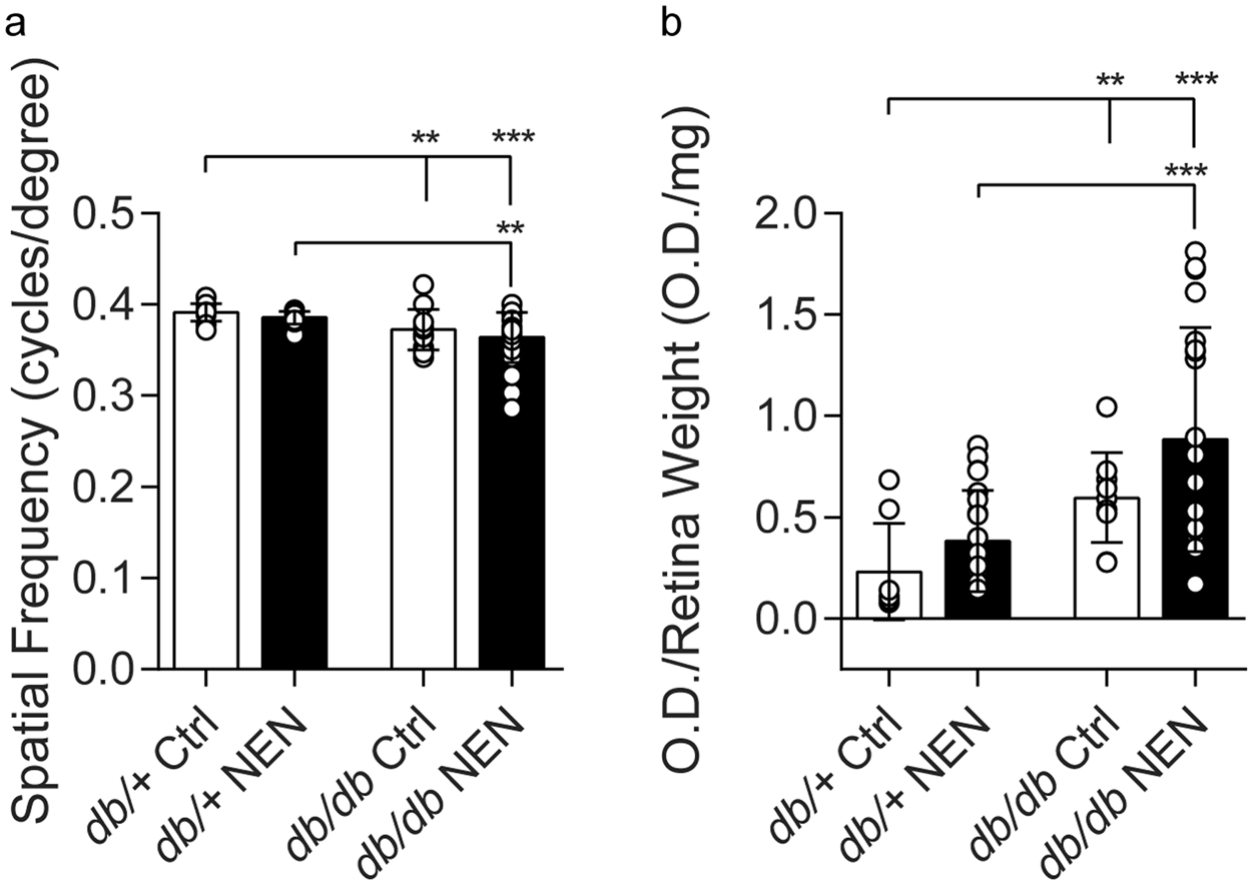
Diabetic Retinopathy Phenotyping. (**a**) Visual acuity, and (**b**) retinal DNA fragmentation were measured at 24 wk. Data are mean ± SD from individual eyes. *p < 0.05, **p < 0.01, ***p < 0.001.

Similarly, we confirmed that *db/db* mice developed features of early stage DR, with compromised visual performance, as measured by visual acuity testing (Fig. 5a, p = 0.0041, *db/db* Ctrl vs. *db*/+ Ctrl), and increased retinal apoptosis (Fig. 5b, p = 0.0013, *db/db* Ctrl vs. *db*/+ Ctrl). Again, NEN-treated *db/db* mice did not show any improvement in DR measures.

In summary, *db/db* mice developed renal dysfunction, renal pathology, compromised visual acuity, and retinal neurodegeneration. NEN treatment did not prevent these *db/db* phenotypes.

### Mitochondrial coupling efficiency in dorsal root ganglion neurons

**Figure 6:**
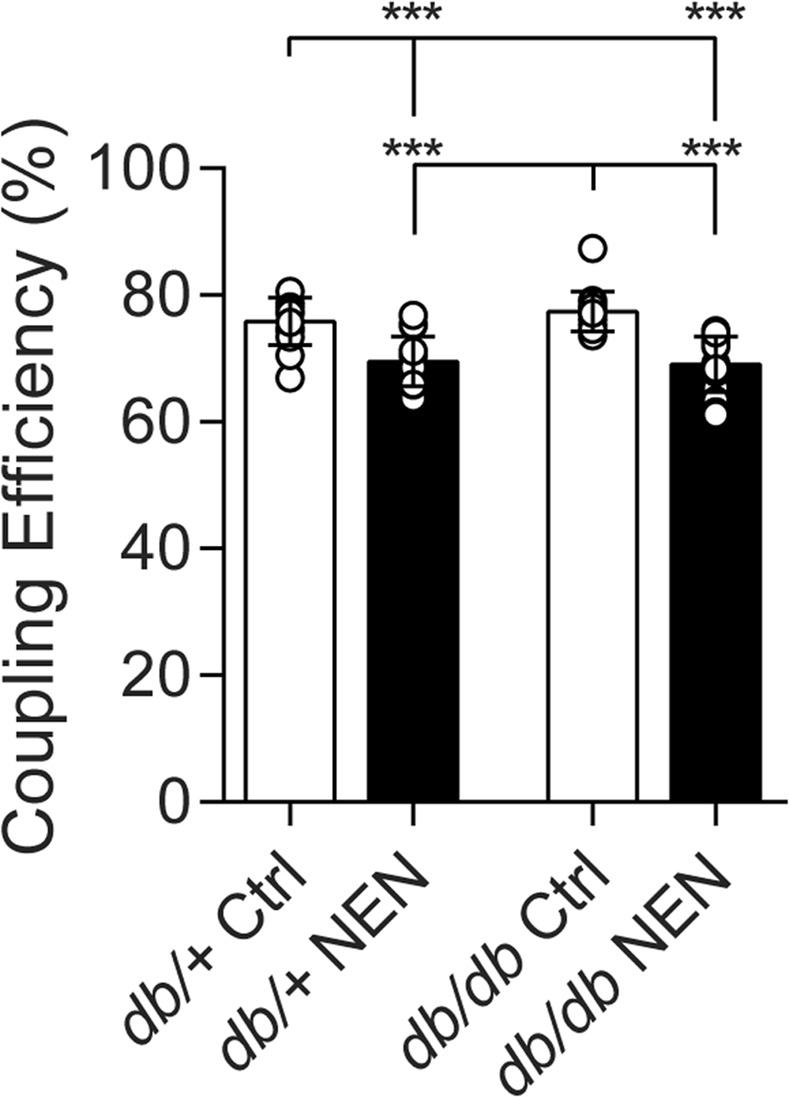
DRG neuron mitochondrial coupling efficiency. Resting mitochondrial coupling efficiency of primary cultured DRG neurons was determined via Seahorse XF Analyzer at 24 wk. Replicates with raw baseline OCR < 50 pmol/min were excluded from analyses according to manufacturer’s recommendations. Data are reported as mean of all replicates. Ctrl, mice fed a standard diet; NEN, mice fed niclosamide ethanolamine chow. *p < 0.05, **p < 0.01, ***p < 0.001.

To determine whether NEN was uncoupling mitochondria in complications-prone tissues, we assessed mitochondrial coupling efficiency in DRG neurons cultured from all groups at 24 wk. Using the Seahorse XF Analyzer, we determined that 75.9% of the oxygen consumed by mitochondria was coupled to ATP production in neurons from *db*/+ Ctrl mice, with this coupling efficiency maintained in *db/db* Ctrl neurons (77.4%) (Fig. 6). Mitochondria were significantly uncoupled in neurons from NEN-treated mice, with approximately 69% coupling efficiency in both *db*/+ and *db/db* neurons (p = 0.0002, *db*/+ NEN vs. *db*/+ Ctrl; p < 0.0001, *db/db* NEN vs. *db/db* Ctrl). These data confirm that NEN was delivered to the mice and that NEN uncoupled extrahepatic tissues.

## Discussion

We recently identified fundamental differences in mitochondrial responses of peripheral nerve, kidney, and retinal tissues to T2D in BKS-*db/db* mice^10,13^. However, whether these mitochondrial adaptations are the cause or consequence of tissue dysfunction remains unclear. In the current study BKS-*db/db* mice were treated with the mitochondrial uncoupler, NEN, beginning at 6 wk of age to determine the effects of mitochondrial uncoupling therapy on T2D, and the pathogenesis of DPN, DKD and DR. We anticipated that NEN therapy would provide therapeutic benefit by either directly improving the T2D phenotype, or via direct action on mitochondria in the complications-prone tissues. We report that NEN treatment from 6–24 wk of age had little effect on the development of T2D and diabetic complications.

While uncoupling therapy is reported to improve glycemic control in T2D^16^, we observed no improvement in glycemia with NEN therapy beginning at 6 wk of age and continuing through 24 wk. Interestingly, this could be due to the fact that the drug was not introduced until 6 wk of age. When begun in the same BKS-*db/db* model at 5 wk of age and continued until 13 wk, NEN treatment modestly, but significantly, decreased glycated hemoglobin^16^. This idea of “age dependence” is further supported by data from another uncoupler, MitoQ. When MitoQ was administered in the BKS-*db/db* mice from 8 wk of age and continued to 20 wk, there was no improvement in glycemia^37^. Collectively, these findings suggest that the aggressive progression of severe hyperglycemia in *db/db* mice^34^ creates a short, very early time window for therapeutic intervention for uncoupling drugs. This is also supported by the fact that NEN and its parent compound, niclosamide, improve glycemic control in a murine model that exhibits only a modest increase in glucose (high fat diet-fed mouse model of obesity and impaired glucose tolerance)^16,38^.

Although *db/db* mice were consistently obese, we observed a body weight plateau, with subsequent weight loss. These longitudinal changes are common in BKS-*db/db* mice^16,39^, likely related to beta cell decompensation^39^. We observed that NEN-*db/db* mice were heavier than *db/db* mice during this plateau phase (12–18 wk of age/6–12 wk of NEN treatment), results that confirm a previous report of maintained higher body weights in NEN-*db/db* mice compared with age-matched *db/db* controls (9–13 wk of age/4–8 wk of NEN treatment)^16^. This is an important finding, as it confirms the bioactivity of NEN in the current study. Importantly, niclosamide salt derivatives, NEN and niclosamide piperazine, decrease body weight in high fat diet-fed C57BL/6 J mice^16,40^, suggesting that NEN effects on weight in *db/db* mice may be specific to this model with its genetic disruption of the leptin signaling pathway. An alternative idea lies in the fact that NEN is reported to primarily target hepatic lipid metabolism^16^. It is well established that liver pathology is less severe in BKS-*db/db* mice than C57BL/6 J mice fed a high fat diet^41,42^. BKS-*db/db* mice develop macrovesicular hepatic steatosis, but unlike high fat fed C57BL/6 J mice, they do not develop fibrosis or nonalcoholic steatohepatitis (NASH)^41,42^. It could be that NEN is more effective in models with a more severe liver phenotype; this idea could not only explain the NEN-mediated changes in weight, but could also contribute to the reported improved glycemia in selected murine models^16^.

Cholesterol profiles are not frequently reported in the uncoupling literature, however, our observation that NEN treatment significantly lowered free cholesterol in *db/db* mice is consistent with reports that niclosamide and a 2,4-dinitrophenol (DNP) derivative decrease both cholesterol^38,43^ and LDL-cholesterol^38^ in high fat diet-fed mice. To our knowledge, only two studies have investigated uncoupling therapies in *db/db* mice^16,37^, and neither of these studies assessed cholesterol levels. We observed no functional consequence of lowered cholesterol, although these findings, again, confirmed drug bioactivity.

We had speculated that even if we did not observe an effect on glycemia, there could be tissue-specific effects of NEN in each of the diabetic complications: DPN, DKD and DR. We were particularly interested in DPN, as we had previously shown that overexpression of uncoupling proteins could block hyperglycemia-induced injury of primary sensory neurons in culture^17^. There is also a report that niclosamide attenuates mechanical hyperalgesia (a measure of sensory Aβ fibers) in the partial sciatic nerve ligation rat model of neuropathic pain^44^. Despite these encouraging *in vitro* and *in vivo* data, we observed no effect of NEN on hind paw withdrawal latencies and IENFD (measures of sensory Aδ and C fibers^45,46^), or large nerve fiber function, as measured by nerve conduction studies. Differences in experimental models and assessment of different populations of peripheral nerve fibers may contribute to these discrepancies, but in our study, NEN had no beneficial effect on DPN.

The lack of a therapeutic effect of NEN on DPN, lead us to assess mitochondrial coupling efficiency in the sensory neurons. Mitochondrial uncoupling was not observed in *db/db* neurons cultured from these mice, consistent with previous reports in DRG neurons from STZ-T1D rats^47^. We anticipated that if the decreased mitochondrial lipid flux previously observed in the *db/db* peripheral nerve^13^ was in itself responsible for mitochondrial dysfunction, uncoupling therapy would increase flux (Fig. 1a), and improve DPN by reversing mitochondrial dysfunction. The fact that we observed NEN uncoupling of neuronal mitochondria with no change in DPN phenotype suggests that mild uncoupling of neuronal mitochondria is neither harmful nor beneficial to peripheral nerves. This was unexpected as we previously reported that direct modulation of the mitochondrial coupling/uncoupling balance via *Ucp3* overexpression prevented glucose-induced mitochondrial membrane depolarization and programmed cell death in primary cultured neurons^17^. Notably, these *in vitro* overexpression experiments were performed on embryonic DRG neurons (known to have different metabolic requirements than adult DRG neurons^48^) in response to acute, 6 h high glucose conditions. The role of lipids was not investigated, nor did these experiments account for longitudinal responses to the dynamic and complex *in vivo* T2D environment. Moreover, while NEN-induced uncoupling in the current study was significant, it was mild, suggesting that a more pronounced neuronal uncoupling may be required to affect DPN phenotypes.

As mitochondrial dysfunction is reported to be a precipitating event in DKD in T1D rats^49^, we expected NEN therapy to affect DKD pathogenesis. MitoQ-mediated uncoupling therapy improves albuminuria and glomerular filtration rate in *db/db* mice (from 8–20 wk), despite not improving glycemic control or body weight^37^. Therefore, although we saw no improvements in glycemia or body weight with NEN, we may have expected to see a positive impact of NEN on DKD. However, similar to DPN, we observed no effect of NEN treatment on DKD phenotype. Interestingly, genetic deletion of *Ucp2* prevents proteinuria in T1D mice^20^. This suggests that the consistently observed UCP2 upregulation and mitochondrial uncoupling in both T1D and T2D kidneys^13,18–23^ likely contributes to early DKD pathogenesis. We therefore could have equally expected NEN uncoupling treatment to exacerbate DKD in the current study, a finding we observed, although it did not reach statistical significance.

Polymorphisms in retinal *UCP1* and *UCP2* are associated with DR in T1D and T2D in humans^25,50^. Moreover, increased whole-retina UCP2 activity in STZ-T1D rats is reported to be protective, limiting production of reactive oxygen species and maintaining ATP production^24^. Although these data suggest that regulation of mitochondrial coupling/uncoupling responses are important in retina, similar to DPN and DKD, we did not see a significant effect of NEN treatment on DR pathogenesis.

In short, the lack of NEN treatment effect on diabetic complications was unexpected. Our results suggest that regulation of mitochondrial coupling/uncoupling within a tissue is specific, finely balanced, and likely changes in response to the dynamic T2D disease course. While it is well-established that differential regulation of uncoupling proteins (UCPs) is associated with DPN^17^, DKD^13,18–23^, and DR^24–26^, these studies involved large changes in endogenous UCP expression and function (overexpression or complete knockout). The three most widely investigated UCPs (UCP1–3) have overlapping and differential functions, respond to different physiological stimuli, and have tissue-specific expression^51^. Specifically, kidney predominantly expresses UCP2^13,18,19,21,22^, retina expresses UCP1 and UCP2^24–26^, while DRG neurons express UCP3^17^. Moreover, differential effects of UCP2 inhibition have been observed within the same tissue: improving kidney phenotypes in some paradigms^21,22^, but worsening phenotypes in others^23^. Perhaps a titrated and specifically-targeted uncoupling therapy may have greater efficacy in treating diabetic complications, but our results show a pan-uncoupling approach is not likely to provide therapeutic benefit for all three diabetic complications.

In summary, we report that NEN itself is not injurious (no effect on *db*/+ control mice) and conclude that globally targeting mitochondria with an uncoupling drug is unlikely to provide therapeutic benefit for DPN, DKD, or DR. Our findings also highlight the need for further insights into the role of tissue-specific metabolic reprogramming in the pathogenesis of diabetic microvascular complications.

## Methods

### Animal model & Study design

Twenty-four male BKS *db*/+ (control) and 24 male *db/db* (diabetic) mice (BKS.Cg-m +/+ Lepr^db^/J; stock number 000642, Jackson Laboratory, Bar Harbor, ME) were purchased at 5 wk of age. All mice were fed a standard diet (AIN-93M chow, #D10012M, Research Diets, New Brunswick, NJ) for a 1-week acclimation period prior to random cage assignment to control or NEN treatment groups. Control mice continued on the standard diet, and NEN mice were fed standard diet supplemented with 1500 ppm NEN from 6 wk of age (#D11070502, Research Diets, New Brunswick, NJ) (Fig. 1b). At study termination, mice were euthanized with 150 mg/kg of pentobarbital (i.p.). Blood was immediately collected via the superior vena cava for glycated hemoglobin (%HbA1c) and plasma processing. Hind feet were removed for intraepidermal nerve fiber counts, prior to systemic perfusion with ~30 mL PBS via the left ventricle. Animals were maintained in a pathogen-free environment and cared for by the University of Michigan (U-M) Unit for Laboratory Animal Medicine. All protocols followed the Diabetic Complications Consortium guidelines (www.diacomp.org) and were approved by the U-M University Committee on Use and Care of Animals. The datasets generated and analyzed during the current study are available from the corresponding author on reasonable request.

### Metabolic phenotyping

Body weights were measured every 2 wk, from 12–24 wk. Four h fasting blood glucose (FBG) was measured from tail-blood using an AlphaTrak Glucometer (Abbott Laboratories, Abbott Park, IL). FBG was measured at 16, 20 and 24 wk. Terminal glycated hemoglobin (%HbA1c), and free plasma cholesterol and cholesterol esters were measured via ELISA, according to manufacturer’s protocols (Mouse HbA1c Assay Kit #80310, CrystalChem, Elk Grove Village, IL) (Cholesterol Assay Kit #ab65390, Abcam, Cambridge, MA). Total plasma cholesterol was measured by the Michigan Diabetes Research Center (University of Michigan, Ann Arbor, MI). Total plasma triglycerides were measured by the Mouse Metabolic Phenotyping Core (www.mmpc.org).

### Diabetic complications phenotyping

Peripheral nerve, kidney, and retina phenotyping was performed according to Diabetic Complications Consortium guidelines (www.diacomp.org/shared/protocols.aspx), as described below.

### Diabetic peripheral neuropathy phenotyping

Sensory and motor large fiber function, small fiber function, and small fiber loss were determined at 24 wk of age according to our previously published protocols^52–55^.

#### Hind paw withdrawal latency

Small nocifensive fiber function was assessed via withdrawal latency from a thermal stimulus. Mice were placed in the thermal testing apparatus maintained at 30 °C and allowed to habituate for 45 min. The infrared heat source (Model 336TG Life Sciences, Woodland Hills, CA) was positioned under the plantar surface of the hind paw, and the elapsed time between stimulus activation and paw withdrawal was recorded. The infrared heat source was set at 30 °C and the temperature increased over the course of 20 s. A 20 s time threshold was set to prevent injury to the mice. The approximate maximum withdrawal response occurred at 60 °C. Six measurements were obtained per mouse, 3 from each foot, with the average being taken as the final withdrawal latency.

#### Nerve conduction velocities (NCVs)

Large nerve fiber function was assessed via sural sensory NCV (sNCV), and sciatic motor NCV (mNCV) electrophysiological testing. Measurements were performed using stainless steel needle electrodes (Natus Biomedical, Madison, WI), under 1–2% isoflurane^52^, with body temperature maintained at 34 °C with a heating lamp. Sural sensory NCV was determined by recording at the dorsum of the foot and applying antidromic, supramaximal stimulation at the ankle. The NCV was calculated by dividing the distance by the take-off latency of the sensory nerve action potential. Sciatic-tibial motor NCV was determined by recording at the dorsum of the foot and applying orthodromic, supramaximal stimulation at the ankle, then at the sciatic notch. Latencies were measured in each case from the initial onset of the compound muscle action potential. The motor NCV was calculated by subtracting the measured ankle distance from the measured notch distance. The resultant distance was then divided by the difference in the ankle and notch latencies for a final nerve conduction velocity.

#### Intraepidermal nerve fiber counts

Cutaneous small fiber nerve degeneration was assessed via intraepidermal nerve fiber density (IENFD) profiles. Prior to systemic PBS perfusion, foot pads were collected from the plantar surface of the hind paw, immersed in Newcomer Zamboni’s fixative (Middletown, WI) overnight at 4 °C, cryoprotected overnight at 4 °C in 30% sucrose in 0.1 M sodium phosphate buffer, cryoembedded, sectioned (30 μm) and processed for pan-axonal marker, PGP9.5, immunofluorescence (1:2000 Proteintech, Rosemont, IL). Three images per mouse (3 mm) were collected on an Olympus FluoView 500 confocal microscope using a 20 × 1.2 objective at a resolution of 1024 × 1024 pixels. The optical section thickness was 3.3 μm. Ten images per stack were flattened using max project arithmetic option in MetaMorph (version 7.7.0.00, Molecular Devices). Counts and distances were summed, and the data are presented as the number of fibers per millimeter.

### Diabetic kidney disease phenotyping

Changes in kidney function and structure were determined at 24 wk of age using our published protocols^56,57^.

#### Metabolic urine

Terminal urine samples were collected in murine metabolic cages (Hatteras Instruments, Cary, NC) over the final 24 h of a 72 h period^5^. Food and water were available *ad libitum*. Urinary albumin and creatinine levels were determined using the Albuwell M and Companion Creatinine systems (Exocell, Philadelphia, PA) according to Diabetic Complications Consortium protocols (https://www.diacomp.org/shared/protocols.aspx).

#### Glomerular hypertrophy and mesangial index

Following systemic PBS perfusion, the left kidney was removed, weighed, and fixed overnight in 2% paraformaldehyde in PBS. Kidneys were paraffin-embedded, sectioned (3 µm), and stained with periodic acid-Schiff (PAS) reagent^56^. Fifteen glomerular tufts per animal were randomly selected for analysis. Mesangial area was quantified by calculating the percentage of the total glomerular area that was PAS-positive. Quantification was performed with MetaMorph (version 6.14), and microscope images captured using a digital camera.

### Diabetic retinopathy phenotyping

Visual performance and retinal degeneration were determined at 24 wk of age according to our previously published protocols^58,59^.

#### Optokinetic tracking

Visual acuity was assessed via a virtual-reality optokinetic tracking system (OptoMotry, CerebralMechanics, Inc., Alberta, Canada)^58^ by the Visual Funct Assessment Module of the University of Michigan Kellogg Eye Center Vision Core. Mice were placed, unrestrained, on a pedestal inside a chamber containing four computer monitors projecting a moving visual grating stimulus in 3-dimensional space, composed of an alternating rotating sine wave with 100% contrast. The mouse head movements were tracked, in a temporal to nasal direction and a simple staircase method was employed to identify the highest grade of spatial frequency (“acuity”) visible to the animal. Beginning with a spatial frequency of 0.042 cycles/degree, assessments were completed with a drift speed of 12 degrees/s.

#### Retinal DNA fragmentation

Retinal degeneration was assessed via apoptotic DNA cleavage ELISA (Cell Death Detection, Roche Applied Science, Indianapolis, IN) exactly as previously described^59^. Relative DNA fragmentation was expressed as optical density (light absorbance at 405 nm, with a 490 nm reference wavelength), normalized to retinal wet weight.

#### Dorsal root ganglion neuron culture and mitochondrial coupling efficiency

Cervical, lumbar, and thoracic dorsal root ganglia (DRG) from 2 mice per group were dissociated and DRG neurons cultured on lamnin-coated Seahorse XF24 microplates (Agilent Technologies, Santa Clara, CA) according to our published protocols^60,61^. DRG neurons were plated in *plating media* (20 wells/group), switched to *feed media* after 24 h, switched to *treatment media* after another 24 h (48 h total), and coupling efficiency assessed after another 24 h (72 h total). *Treatment media:* 50% F-12K, 50% DMEM, 1:100 dilution of Nb+, 1000 U/ml penicillin/streptomycin/neomycin, and 7.2 μM aphidicolin. *Feed media*: treatment medium plus 1×B27. *Plating media:* feed medium plus 2 mM l-glutamine (0.4 μM final concentration). Mitochondrial coupling efficiency was assessed using the Seahorse XF24 Analyzer (Agilent Technologies, Santa Clara, CA, USA). XF Analyzers use electro-optical technology for measurement of real-time rates of oxygen consumption (OCR). Following stable baseline OCR, mitochondrial respiratory chain inhibitors, oligomycin and antimycin A, were sequentially added, and subsequent changes in OCR were used to determine mitochondrial coupling efficiency as previously described^62^. Replicates with raw baseline OCR < 50 pmol/min were excluded from analyses according to manufacturer’s recommendations. Data are reported as mean of all replicates.

### Statistical analysis

Analyses were performed using GraphPad Prism 7, according to Festing & Altman^63^. Normality of data was determined using Brown-Forsythe F-tests. For normally distributed data, statistically significant differences (p < 0.05) were determined using one-way ANOVA with Tukey’s post-test for multiple comparisons. For non-normally distributed data, datasets were log2 transformed and the Brown-Forsythe F-test re-run. When log2-transformation normalized distribution, a one-way ANOVA with Tukey’s post-test for multiple comparisons was run. When log2-transformation did not normalize distribution, the non-parametric Kruskal-Wallis test, with Dunn’s post-test for multiple comparisons was run on the original, non-transformed dataset. Data are presented as mean ± standard deviation. Individual analyses and P values for all dataset comparisons are provided in Supplementary data file 1.

## Supplementary information

Supplementary Information

Supplementary data file 1

## Data Availability

All data generated or analyzed during this study are included in this published article (and its Supplementary files).

## References

[1] International Diabetes Federation. IDF Diabetes Atlas, 6th edn. Brussels, Belgium: *International Diabetes Federation* http://www.idf.org/diabetesatlas (2013).

[2] Bullock A, Sheff K (2017). Incidence Trends of Type 1 and Type 2 Diabetes among Youths, 2002-2012. N Engl J Med.

[3] Jensen ET, Dabelea D (2018). Type 2 Diabetes in Youth: New Lessons from the SEARCH Study. Current diabetes reports.

[4] Callaghan BC, Price RS, Feldman EL (2015). Distal Symmetric Polyneuropathy: A Review. JAMA.

[5] Afkarian M (2016). Clinical Manifestations of Kidney Disease Among US Adults With Diabetes, 1988-2014. JAMA.

[6] Lee R, Wong TY, Sabanayagam C (2015). Epidemiology of diabetic retinopathy, diabetic macular edema and related vision loss. Eye Vis (Lond).

[7] Edwards JL, Vincent AM, Cheng HT, Feldman EL (2008). Diabetic neuropathy: mechanisms to management. Pharmacol Ther.

[8] Feldman EL (2008). Diabetic neuropathy. Curr Drug Targets.

[9] Flaxman SR (2017). Global causes of blindness and distance vision impairment 1990-2020: a systematic review and meta-analysis. Lancet Glob Health.

[10] Hinder LM (2017). Comparative RNA-Seq transcriptome analyses reveal distinct metabolic pathways in diabetic nerve and kidney disease. J Cell Mol Med.

[11] Hur J (2016). Transcriptional networks of murine diabetic peripheral neuropathy and nephropathy: common and distinct gene expression patterns. Diabetologia.

[12] Sas KM (2018). Shared and distinct lipid-lipid interactions in plasma and affected tissues in a diabetic mouse model. J Lipid Res.

[13] Sas KM (2016). Tissue-specific metabolic reprogramming drives nutrient flux in diabetic complications. JCI Insight.

[14] Leverve XM (2007). Mitochondrial function and substrate availability. Crit Care Med.

[15] Skulachev VP (1998). Uncoupling: new approaches to an old problem of bioenergetics. Biochim Biophys Acta.

[16] Tao H, Zhang Y, Zeng X, Shulman GI, Jin S (2014). Niclosamide ethanolamine-induced mild mitochondrial uncoupling improves diabetic symptoms in mice. Nat Med.

[17] Vincent AM, Olzmann JA, Brownlee M, Sivitz WI, Russell JW (2004). Uncoupling proteins prevent glucose-induced neuronal oxidative stress and programmed cell death. Diabetes.

[18] Friederich M, Fasching A, Hansell P, Nordquist L, Palm F (2008). Diabetes-induced up-regulation of uncoupling protein-2 results in increased mitochondrial uncoupling in kidney proximal tubular cells. Biochim Biophys Acta.

[19] Persson MF (2012). Coenzyme Q10 prevents GDP-sensitive mitochondrial uncoupling, glomerular hyperfiltration and proteinuria in kidneys from db/db mice as a model of type 2 diabetes. Diabetologia.

[20] Friederich-Persson, M., Persson, P., Hansell, P. & Palm, F. Deletion of Uncoupling Protein-2 reduces renal mitochondrial leak respiration, intrarenal hypoxia and proteinuria in a mouse model of type 1 diabetes. *Acta physiologica (Oxford, England)*, e13058, 10.1111/apha.13058 (2018). 10.1111/apha.13058 29480974

[21] Friederich-Persson M (2012). Acute knockdown of uncoupling protein-2 increases uncoupling via the adenine nucleotide transporter and decreases oxidative stress in diabetic kidneys. PLoS One.

[22] Qiu W (2012). Genipin inhibits mitochondrial uncoupling protein 2 expression and ameliorates podocyte injury in diabetic mice. PLoS One.

[23] Chen XL, Tang WX, Tang XH, Qin W, Gong M (2014). Downregulation of uncoupling protein-2 by genipin exacerbates diabetes-induced kidney proximal tubular cells apoptosis. Renal failure.

[24] Osorio-Paz I, Uribe-Carvajal S, Salceda R (2015). In the Early Stages of Diabetes, Rat Retinal Mitochondria Undergo Mild Uncoupling due to UCP2 Activity. PLoS One.

[25] Brondani LA (2012). The UCP1 -3826A/G polymorphism is associated with diabetic retinopathy and increased UCP1 and MnSOD2 gene expression in human retina. Invest Ophthalmol Vis Sci.

[26] Cui Y (2006). Expression modification of uncoupling proteins and MnSOD in retinal endothelial cells and pericytes induced by high glucose: the role of reactive oxygen species in diabetic retinopathy. Exp Eye Res.

[27] O’Brien, P. D., Sakowski, S. A. & Feldman, E. L. Mouse models of diabetic neuropathy. *ILAR J*. (2013). 10.1093/ilar/ilt052 PMC3962259 24615439

[28] Sullivan KA (2007). Mouse models of diabetic neuropathy. Neurobiol Dis.

[29] Brosius FC, Alpers CE (2013). New targets for treatment of diabetic nephropathy: what we have learned from animal models. Current opinion in nephrology and hypertension.

[30] Bogdanov P (2014). The db/db mouse: a useful model for the study of diabetic retinal neurodegeneration. PLoS One.

[31] Hernandez C (2017). Topical administration of DPP-IV inhibitors prevents retinal neurodegeneration in experimental diabetes. Diabetologia.

[32] Liu Y (2017). Sensory and autonomic function and structure in footpads of a diabetic mouse model. Sci Rep.

[33] Hummel KP, Dickie MM, Coleman DL (1966). Diabetes, a new mutation in the mouse. Science.

[34] O’Brien PD, Sakowski SA, Feldman EL (2014). Mouse models of diabetic neuropathy. ILAR J.

[35] Hinder LM (2018). Transcriptional networks of progressive diabetic peripheral neuropathy in the db/db mouse model of type 2 diabetes: An inflammatory story. Exp Neurol.

[36] Hur J (2015). The Metabolic Syndrome and Microvascular Complications in a Murine Model of Type 2 Diabetes. Diabetes.

[37] Ward MS (2017). Targeted mitochondrial therapy using MitoQ shows equivalent renoprotection to angiotensin converting enzyme inhibition but no combined synergy in diabetes. Sci Rep.

[38] Al-Gareeb AI, Aljubory KD, Alkuraishy HM (2017). Niclosamide as an anti-obesity drug: an experimental study. Eat Weight Disord.

[39] Dalboge LS (2013). Characterisation of age-dependent beta cell dynamics in the male db/db mice. PLoS One.

[40] Guo, J., Tao, H., Alasadi, A., Huang, Q. & Jin, S. Niclosamide piperazine prevents high-fat diet-induced obesity and diabetic symptoms in mice. *Eat Weight Disord*, 10.1007/s40519-017-0424-7 (2017). 10.1007/s40519-017-0424-7 28780747

[41] Sanches SC, Ramalho LN, Augusto MJ, da Silva DM, Ramalho FS (2015). Nonalcoholic Steatohepatitis: A Search for Factual Animal Models. Biomed Res Int.

[42] Van Herck, M. A., Vonghia, L. & Francque, S. M. Animal Models of Nonalcoholic Fatty Liver Disease-A Starter’s Guide. *Nutrients* **9**, 10.3390/nu9101072 (2017). 10.3390/nu9101072 PMC5691689 28953222

[43] Wei G, Song X, Fu Y, Gong T, Zhang Q (2017). Sustained-release mitochondrial protonophore reverses nonalcoholic fatty liver disease in rats. Int J Pharm.

[44] Ai, N., Wood, R. D., Yang, E. & Welsh, W. J. Niclosamide is a Negative Allosteric Modulator of Group I Metabotropic Glutamate Receptors: Implications for Neuropathic Pain. *Pharm Res* **33**, 3044–3056, 10.1007/s11095-016-2027-9 (2016). 10.1007/s11095-016-2027-9 27631130

[45] Chen YS, Chung SS, Chung SK (2005). Noninvasive monitoring of diabetes-induced cutaneous nerve fiber loss and hypoalgesia in thy1-YFP transgenic mice. Diabetes.

[46] Lennertz RC, Medler KA, Bain JL, Wright DE, Stucky CL (2011). Impaired sensory nerve function and axon morphology in mice with diabetic neuropathy. J Neurophysiol.

[47] Roy Chowdhury SK (2012). Impaired adenosine monophosphate-activated protein kinase signalling in dorsal root ganglia neurons is linked to mitochondrial dysfunction and peripheral neuropathy in diabetes. Brain.

[48] Melli G, Hoke A (2009). Dorsal Root Ganglia Sensory Neuronal Cultures: a tool for drug discovery for peripheral neuropathies. Expert Opin Drug Discov.

[49] Coughlan MT (2016). Mapping time-course mitochondrial adaptations in the kidney in experimental diabetes. Clin Sci (Lond).

[50] Crispim D (2010). Polymorphisms of the UCP2 gene are associated with proliferative diabetic retinopathy in patients with diabetes mellitus. Clin Endocrinol (Oxf).

[51] Sivitz WI, Yorek MA (2010). Mitochondrial dysfunction in diabetes: from molecular mechanisms to functional significance and therapeutic opportunities. Antioxid Redox Signal.

[52] Oh SS, Hayes JM, Sims-Robinson C, Sullivan KA, Feldman EL (2010). The effects of anesthesia on measures of nerve conduction velocity in male C57Bl6/J mice. Neurosci Lett.

[53] Cheng HT, Dauch JR, Hayes JM, Hong Y, Feldman EL (2009). Nerve growth factor mediates mechanical allodynia in a mouse model of type 2 diabetes. J Neuropathol Exp Neurol.

[54] Cheng HT, Dauch JR, Hayes JM, Yanik BM, Feldman EL (2012). Nerve growth factor/p38 signaling increases intraepidermal nerve fiber densities in painful neuropathy of type 2 diabetes. Neurobiol Dis.

[55] Hinder LM (2017). Dietary reversal of neuropathy in a murine model of prediabetes and the metabolic syndrome. Dis Model Mech.

[56] Zhang H (2008). Rosiglitazone reduces renal and plasma markers of oxidative injury and reverses urinary metabolite abnormalities in the amelioration of diabetic nephropathy. Am J Physiol Renal Physiol.

[57] Sanden SK, Wiggins JE, Goyal M, Riggs LK, Wiggins RC (2003). Evaluation of a thick and thin section method for estimation of podocyte number, glomerular volume, and glomerular volume per podocyte in rat kidney with Wilms’ tumor-1 protein used as a podocyte nuclear marker. Journal of the American Society of Nephrology: JASN.

[58] Wubben TJ (2017). Photoreceptor metabolic reprogramming provides survival advantage in acute stress while causing chronic degeneration. Sci Rep.

[59] Abcouwer SF (2010). Effects of ischemic preconditioning and bevacizumab on apoptosis and vascular permeability following retinal ischemia-reperfusion injury. Invest Ophthalmol Vis Sci.

[60] Vincent AM (2010). Mitochondrial biogenesis and fission in axons in cell culture and animal models of diabetic neuropathy. Acta Neuropathol.

[61] Vincent AM (2009). Dyslipidemia-induced neuropathy in mice: the role of oxLDL/LOX-1. Diabetes.

[62] Hinder LM (2014). Long-chain acyl coenzyme A synthetase 1 overexpression in primary cultured Schwann cells prevents long chain fatty acid-induced oxidative stress and mitochondrial dysfunction. Antioxid Redox Signal.

[63] Festing MF, Altman DG (2002). Guidelines for the design and statistical analysis of experiments using laboratory animals. ILAR J.

