# Supplementary material for: Mitochondrial uncoupling has no effect on microvascular complications in type 2 diabetes
Source: Sci Rep. 2019 Jan 29;9:881. doi: 10.1038/s41598-018-37376-y (PMC6351661; doi:10.1038/s41598-018-37376-y)

Mitochondrial uncoupling has no effect on microvascular complications in type 2 diabetes

Supplementary Information

Lucy M. Hinder[1], Kelli M. Sas[2], Phillipe D. O'Brien[1,] Carey Backus[1], Pradeep Kayampilly[2], John M. Hayes[1], Cheng-mao Lin[3], Hongyu Zhang[2], Sumathi Shanmugam[3], Amy E. Rumora[1], Steven F. Abcouwer[3], Frank C. Brosius III[2, 4], Subramaniam Pennathur[2, 4], Eva L. Feldman[1*]

[1]Department of Neurology, University of Michigan, Ann Arbor, MI 48109, USA

[2]Division of Nephrology, Department of Internal Medicine, University of Michigan, Ann Arbor, MI 48109, USA

[3]Department of Ophthalmology and Visual Sciences, University of Michigan, Ann Arbor, Michigan 48109

[4]Departments of Molecular and Integrative Physiology, University of Michigan, Ann Arbor, MI 48109

*****Corresponding author:

Eva L. Feldman, MD, PhD

Russell N. DeJong Professor of Neurology

5017 AATBSRB, 109 Zina Pitcher Place

Ann Arbor, MI 48109, United States

Phone: (734) 763-7274

Fax: (734) 763-7275

Email: efeldman@umich.edu

Supplementary Figures

Supplementary Figure 1. Longitudinal Body Weight and Fasting Blood Glucose. (a) Body weights were measured fortnightly from 12-24 wk. (b) Fasting blood glucose levels were measured at 16, 20 and 24 wk. Ctrl, mice fed a standard diet; NEN, mice fed niclosamide ethanolamine chow. *, p<0.05, **, p<0.01, *db/db* NEN vs. *db/db* Ctrl.

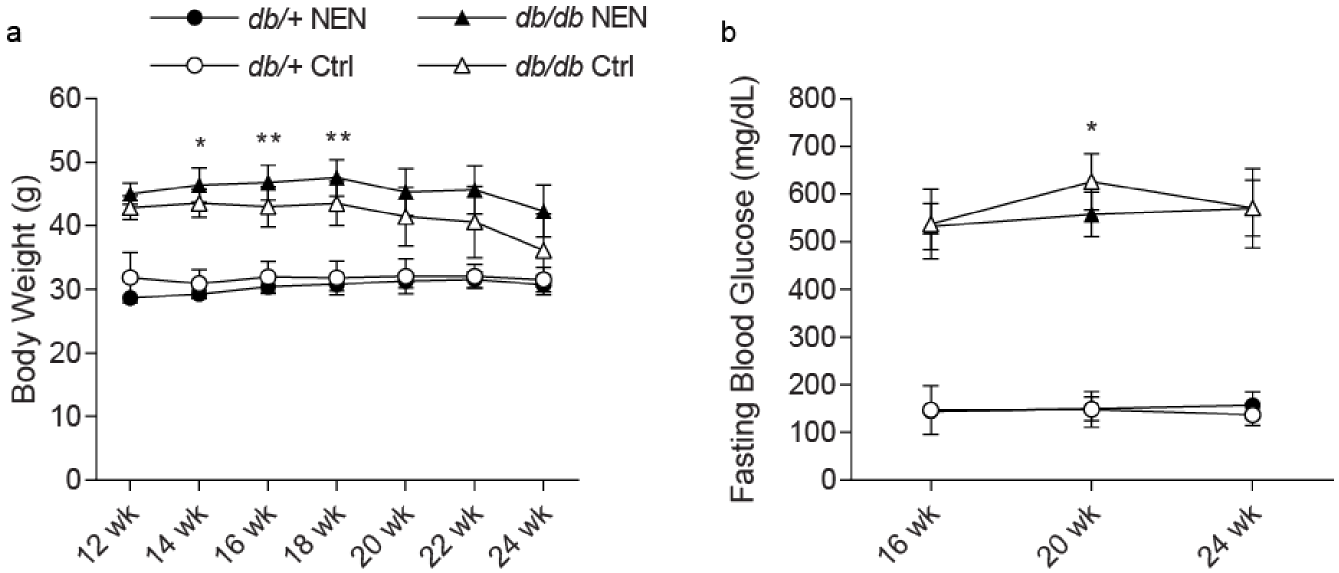

Supplementary Figure 2. Diabetic Kidney Disease (DKD) Phenotyping. (a) 24 h total urinary creatinine, (b) 24 h total urinary albumin, and (c) periodic acid-Schiff (PAS)-positive glomerular area were measured at 24 wk. Ctrl, mice fed a standard diet; NEN, mice fed niclosamide ethanolamine chow. *, p<0.05, ***, p<0.001

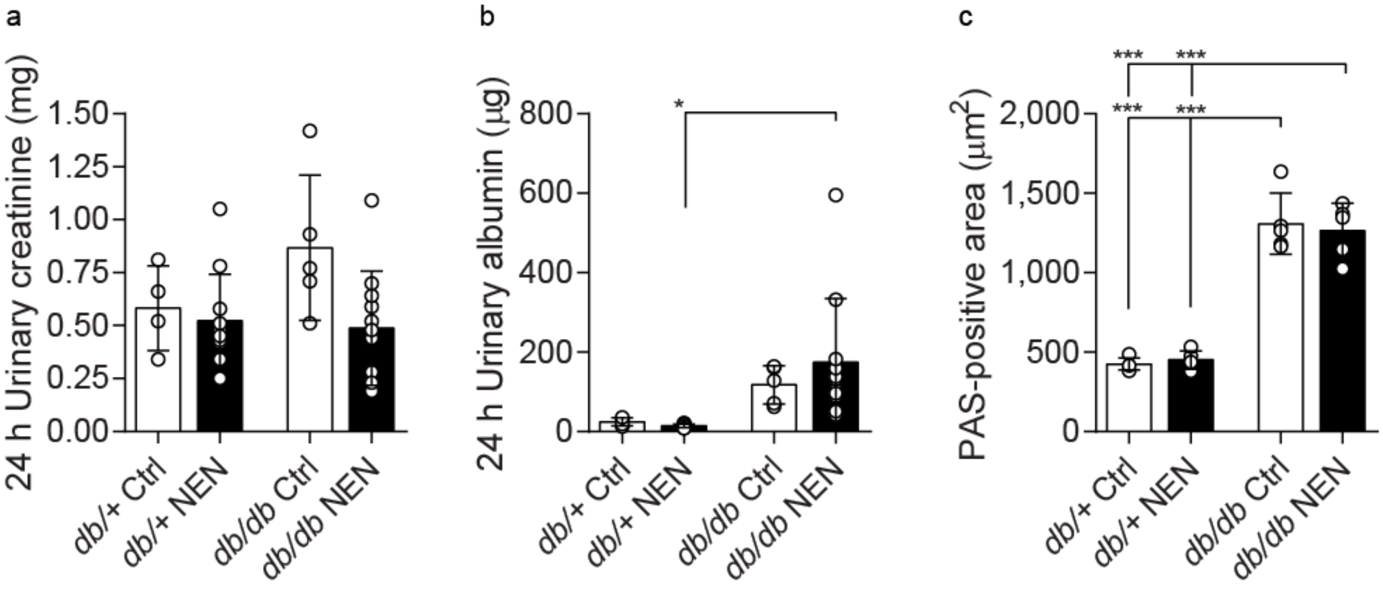

Supplement: Supplementary file 1 — Supplementary Information [file 41598_2018_37376_MOESM1_ESM.pdf]
